# Supplementary material for: SUPREM: an engineered non-site-specific m6A RNA methyltransferase with highly improved efficiency
Source: Nucleic Acids Res. 2024 Oct 17;52(20):12158–72. doi: 10.1093/nar/gkae887 (PMC11551740; doi:10.1093/nar/gkae887)
Supplement: gkae887_Supplemental_Files [file gkae887_supplemental_files.zip › Supplementary Table S2.pdf]

Supplementary Table S2. Sequence of DNA and RNA substrate

Name	Sequence
DNA_substrate_3569bp	TTTCTGCGCGTAATCTGCTGCTTGCAAACAAAAAAACCACCGCTACCAGCGGTGGTTTGTTT<br>GCCGGATCAAGAGCTACCAACTCTTTTTCCGAAGGTAACTGGCTTCAGCAGAGCGCAGATA<br>CCAAATACTGTCCTTCTAGTGTAGCCGTAGTTAGGCCACCACTTCAAGAACTCTGTAGCACC<br>GCCTACATACCTCGCTCTGCTAATCCTGTTACCAGTGGCTGCTGCCAGTGGCGATAAGTCGT<br>GTCTTACCGGGTTGGACTCAAGACGATAGTTACCGGATAAGGCGCAGCGGTCGGGCTGAA<br>CGGGGGGTTCGTGCACACAGCCCAGCTTGGAGCGAACGACCTACACCGAACTGAGATACCT<br>ACAGCGTGAGCTATGAGAAAGCGCCACGCTTCCCGAAGGGAGAAAGGCGGACAGGTATCC<br>GGTAAGCGGCAGGGTCGGAACAGGAGAGCGCACGAGGGAGCTTCCAGGGGGAAACGCCT<br>GGTATCTTTATAGTCCTGTCGGGTTTCGCCACCTCTGACTTGAGCGTCGATTTTTGTGATGCT<br>CGTCAGGGGGGCGGAGCCTATGGAAAAACGCCAGCAACGCGGCCTTTTTACGGTTCCTGG<br>CCTTTTGCTGGCCTTTTGCTCACATGACCCGACACCATCGAATGGCCAGATGATTAATTCCTA<br>ATTTTTGTTGACACTCTATCATTGATAGAGTTATTTTACCACTCCCTATCAGTGATAGAGAAA<br>AGTGAAATGAATAGTTCGACAAAAATCTAGAAATAATTTTGTTTAACTTTAAGAAGGAGATA<br>TACATATGGGCAGCAGCCATCATCATCACCATCATAGCAGCGGCCTGGTGCCGCGCGGCAG<br>CTCCATGTTAAATACAGTAAAAATTTCGTCATGTGAGTTAATCAATGCGGACTGCTTAGAGT<br>TTATCCGCTCTTTGCCGGAAAATTCGGTTGATTTAATTGTTACGGACCCCCCCTACTTTAAGG<br>TAAAACCGGAAGGGTGGGACAACCAGTGGAAGGGCGATGACGACTATTTGAAATGGCTTG<br>ATCAATGTCTGGCACAATTTTGGCGCGTTCTTAAGCCGGCGGGAAGTCTGTACCTTTTTTGC<br>GGTCACCGCTTAGCGTCCGATATCGAGATTATGATGCGCGAACGTTTTTCCGTTTTAAATCA<br>TATTATCTGGGCGAAACCGTCAGGTCGTTGGAACGGGTGCAATAAGGAATCTTTGCGTGCA<br>TACTTTCCCGCGACAGAGCGTATCTTATTTGCGGAACATTATCAGGGGCCATATCGCCCAAA<br>AGATGCTGGTTACGAGGCGAAGGGCCGTGCCCTTAAGCAGCATGTCATGGCTCCTTTGATC<br>GCATACTTCCGTGACGCTCGCGCCGCTCTTGGAATTACCGCAAAGCAAATCGCAGATGCAA<br>CAGGAAAGAAGAACATGGTACCACACTGGTTCTCCGCAAGCCAGTGGCAATTACCCAACGA<br>GTCGGATTACTTGAAATTGCAATCTCTGTTTGCCCGCGTAGCAGAAGAAAAGCACCAACGT<br>GGAGAGCTGGAAAAGCCCCACCATCAGTTGGTTTCTACCTACTCTGAACTTAACCGTAAATA<br>TATGGAATTGTTATCTGAGTATAAAAACCTGCGTCGCTATTTCGGCGTGACAGTGCAAGTCC<br>CATACACGGATGTCTGGACCTACAAGCCTGTACAATATTACCCCGGTAAACACCCCTGCGAA<br>AAACCCGCAGAAATGCTGCAACAAATTATCAGCGCGTCTAGTCGCCCGGGAGACTTGGTTG<br>CAGACTTCTTTATGGGATCGGGCTCTACTGTGAAAGCCGCCATGGCTTTGGGACGCCGCGC<br>GATTGGCGTTGAGTTGGAGACCGGGCGCTTCGAGCAGACTGTACGTGAGGTTCAGGACTT<br>GATCGTCTGATAAGGTAGCGGCCGCTAGCTGCAGCCCGGGATCCGAATTCGAGCTCCGTCG<br>ACAAGCTTGACCTGTGAAGTGAAAAATGGCGCACATTGTGCGACATTTTTTTTGTCTGCCGT<br>TTACCGCTACTGCGTCACGGATCTCCACGCGCCCTGTAGCGGCGCATTAAGCGCGGCGGGT<br>GTGGTGGTTACGCGCAGCGTGACCGCTACACTTGCCAGCGCCCTAGCGCCCGCTCCTTTCGC<br>TTTCTTCCCTTCCTTTCTCGCCACGTTCGCCGGCTTTCCCCGTCAAGCTCTAAATCGGGGGCT<br>CCCTTTAGGGTTCCGATTTAGTGCTTTACGGCACCTCGACCCCAAAAAACTTGATTAGGGTG<br>ATGGTTCACGTAGTGGGCCATCGCCCTGATAGACGGTTTTTCGCCCTTTGACGTTGGAGTCC<br>ACGTTCTTTAATAGTGGACTCTTGTTCCAAACTGGAACAACACTCAACCCTATCTCGGTCTAT<br>TCTTTTGATTTATAAGGGATTTTGCCGATTTCGGCCTATTGGTTAAAAAATGAGCTGATTTAA<br>CAAAAATTTAACGCGAATTTTAACAAAATATTAACGCTTACAATTTCAGGTGGCACTTTTCG<br>GGGAAATGTGCGCGGAACCCCTATTTGTTTATTTTTCTAAATACATTCAAATATGTATCCGCT

	CATGAGACAATAACCCTGATAAATGCTTCAATAATATTGAAAAAGGAAGAGTATGAGTATT<br>CAACATTTCCGTGTCGCCCTTATTCCCTTTTTTGCGGCATTTTGCCTTCCTGTTTTTGCTCACCC<br>AGAAACGCTGGTGAAAGTAAAAGATGCTGAAGATCAGTTGGGTGCACGAGTGGGTTACAT<br>CGAACTGGATCTCAACAGCGGTAAGATCCTTGAGAGTTTTCGCCCCGAAGAACGTTTTCCAA<br>TGATGAGCACTTTTAAAGTTCTGCTATGTGGCGCGGTATTATCCCGTATTGACGCCGGGCAA<br>GAGCAACTCGGTCGCCGCATACACTATTCTCAGAATGACTTGGTTGAGTACTCACCAGTCAC<br>AGAAAAGCATCTTACGGATGGCATGACAGTAAGAGAATTATGCAGTGCTGCCATAACCATG<br>AGTGATAACACTGCGGCCAACTTACTTCTGACAACGATCGGAGGACCGAAGGAGCTAACCG<br>CTTTTTTGCACAACATGGGGGATCATGTAACTCGCCTTGATCGTTGGGAACCGGAGCTGAAT<br>GAAGCCATACCAAACGACGAGCGTGACACCACGATGCCTGTAGCAATGGCAACAACGTTGC<br>GCAAACTATTAACTGGCGAACTACTTACTCTAGCTTCCCGGCAACAATTGATAGACTGGATG<br>GAGGCGGATAAAGTTGCAGGACCACTTCTGCGCTCGGCCCTTCCGGCTGGCTGGTTTATTG<br>CTGATAAATCTGGAGCCGGTGAGCGTGGCTCTCGCGGTATCATTGCAGCACTGGGGCCAGA<br>TGGTAAGCCCTCCCGTATCGTAGTTATCTACACGACGGGGAGTCAGGCAACTATGGATGAA<br>CGAAATAGACAGATCGCTGAGATAGGTGCCTCACTGATTAAGCATTGGTAGGAATTAATGA<br>TGTCTCGTTTAGATAAAAGTAAAGTGATTAACAGCGCATTAGAGCTGCTTAATGAGGTC
RNA_substrate_1872nt	GUCUAGAAAUAAUUUUGUUUAACUUUAAGAAGGAGAUAUAACCAUGAAAAUCGAAGA<br>AGGUAAAGGUCACCAUCACCAUCACCACGGAUCCAUGGAAGACGCCAAAAACAUAAAGA<br>AAGGCCCGGCGCCAUUCUAUCCUCUAGAGGAUGGAACCGCUGGAGAGCAACUGCAUAA<br>GGCUAUGAAGAGAUACGCCCUGGUUCCUGGAACAAUUGCUUUUACAGAUGCACAUAU<br>CGAGGUGAACAUCACGUACGCGGAAUACUUCGAAAUGUCCGUUCGGUUGGCAGAAGC<br>UAUGAAACGAUAUGGGCUGAAUACAAAUCACAGAAUCGUCGUAUGCAGUGAAAACUC<br>UCUUCAAUUCUUUAUGCCGGUGUUGGGCGCGUUAUUUAUCGGAGUUGCAGUUGCGC<br>CCGCGAACGACAUUUAUAAUGAACGUGAAUUGCUCAACAGUAUGAACAUUUCGCAGCC<br>UACCGUAGUGUUUGUUUCCAAAAAGGGGUUGCAAAAAAUUUUGAACGUGCAAAAAAA<br>AUUACCAAUAAUCCAGAAAAUUAUUAUCAUGGAUUCUAAAACGGAUUACCAGGGAUU<br>UCAGUCGAUGUACACGUUCGUCACAUCUCAUCUACCUCCCGGUUUUAAUGAAUACGAU<br>UUUGUACCAGAGUCCUUUGAUCGUGACAAAACAAUUGCACUGAUAAUGAAUUCCUCU<br>GGAUCUACUGGGUUACCUAAGGGUGUGGCCCUUCCGCAUAGAACUGCCUGCGUCAGA<br>UUCUCGCAUGCCAGAGAUCCUAUUUUUGGCAAUCAAAUCAUUCCGGAUACUGCGAUU<br>UUAAGUGUUGUUCCAUUCCAUCACGGUUUUGGAAUGUUUACUACACUCGGAUAUUU<br>GAUAUGUGGAUUUCGAGUCGUCUUAAUGUAUAGAUUUGAAGAAGAGCUGUUUUUAC<br>GAUCCCUUCAGGAUUACAAAAUUCAAAGUGCGUUGCUAGUACCAACCCUAUUUUCAUU<br>CUUCGCCAAAAGCACUCUGAUUGACAAAUACGAUUUAUCUAAUUUACACGAAAUUGCU<br>UCUGGGGGCGCACCUCUUUCGAAAGAAGUCGGGGAAGCGGUUGCAAAACGCUUCCAUC<br>UUCCAGGGAUACGACAAGGAUAUGGGCUCACUGAGACUACAUCAGCUAUUCUGAUUA<br>CACCCGAGGGGGAUGAUAAACCGGGCGCGGUCGGUAAAGUUGUUCCAUUUUUUGAAG<br>CGAAGGUUGUGGAUCUGGAUACCGGGAAAACGCUGGGCGUUAAUCAGAGAGGCGAAU<br>UAUGUGUCAGAGGACCUAUGAUUAUGUCCGGUUAUGUAAACAAUCCGGAAGCGACCA<br>ACGCCUUGAUUGACAAGGAUGGAUGGCUACAUUCUGGAGACAUAGCUUACUGGGACG<br>AAGACGAACACUUCUUCAUAGUUGACCGCUUGAAGUCUUUAAUUAAAUACAAAGGAU<br>AUCAGGUGGCCCCCGCUGAAUUGGAAUCGAUAUUGUUACAACACCCCAACAUCUUCGA<br>CGCGGGCGUGGCAGGUCUUCCCGACGAUGACGCCGGUGAACUUCCCGCCGCCGUUGUU<br>GUUUUGGAGCACGGAAAGACGAUGACGGAAAAAGAGAUCGUGGAUUACGUCGCCAGU

	CAAGUAACAACCGCGAAAAAGUUGCGCGGAGGAGUUGUGUUUGUGGACGAAGUACCGAAAGGUCUUACCGGAAAACUCGACGCAAGAAAAAUCAGAGAGAUCCUCAUAAAGGCCAAGAAGGGCGGAAAGUCCAAACUCGAGUAAGGUUAACCUGCAGGAGGCCUUUAAUUAAGGUGGUGCGGCCGCGCUAGCGGUCCCGGGGGAUCGAUCCGGCUGCUAACAAAGCCCGAAAGGAAGCUGAGUUGGCUGCUGCCACCGCUGAGCAAUAA
RNA_substrate_294nt	GGAAUUGUGAGCGGAUAACAAUUCCCCUCUAGAAAUAAUUUUGUUUAACUUUAAGAAGGAGAUAUACCAUGGGCAGCAGCCAUCAUCAUCAUCAUCACAGCAGCGGCCUGGUGCCGCGCGGCAGCCAUAUGGCUAGCAUGACUGGUGGACAGCAAAUGGGUCGCGGAUCCGAAUUCGAGCUCCGUCGACAAGCUUGCGGCCGCACUCGAGCACCACCACCACCACCACUGAGAUCCGGCUGCUAACAAAGCCCGAAAGGAAGCUGAGUUGGCUGCUGCCACCGCUGAGCAAUAA
