# Supplementary material for: SUPREM: an engineered non-site-specific m6A RNA methyltransferase with highly improved efficiency
Source: Nucleic Acids Res. 2024 Oct 17;52(20):12158–72. doi: 10.1093/nar/gkae887 (PMC11551740; doi:10.1093/nar/gkae887)
Supplement: gkae887_Supplemental_Files [file gkae887_supplemental_files.zip › SupplementaryMethods_Guppy-nanocompore.pdf]

```
#Guppy (version 6.4.2)
#minimap2 (version 2.20)
#samtools (version 1.15.1)
#Nanocompore (version 1.0.4)
#sample1, 2: noMTase-treated RNA
#sample3, 4: M.EcoGII-treated RNA
#sample5, 6: Anc291-treated RNA
#sample7, 8: SUPREM-treated RNA

# Start the Guppy basecaller with specific parameters to create
fastq fikes
guppy_basecaller \
   -i /path/to/data/ \
   --recursive \
   --flowcell FLO-MIN106 --kit SQK-RNA002 \
   --num_callers 16 --device auto \
   --records_per_fastq 8000 \
   -s ./output

# Concatenate the FASTQ files from different samples into single
files
for i in 1 2 3 4 5 6 7 8; do
    cat sample${i}/pass/*.fastq > sample${i}.fastq
done

# Align the reads to the reference genome and sort the output
for i in 1 2 3 4 5 6 7 8; do
    minimap2 -ax map-ont -L /path/to/reference-dna.fa \
    /path/to/sample${i}.fastq | \
    samtools view -bh -F 2324 -q 10 | \
    samtools sort -O bam > sample${i}_aligned_read_bam.fastq;
done

# Index the BAM files
for i in 1 2 3 4 5 6 7 8; do
    samtools index sample${i}_aligned_read_bam.fastq;
done

# Index the FASTQ files using Nanopolish
for i in 1 2 3 4 5 6 7 8; do
    nanopolish index -s /path/to/sequencing_summary.txt \
    -d /path/to/fast5_pass/ ./basecalled_fastq/sample${i}
_aligned_read_bam.fastq;
done

# Perform event alignment using Nanopolish
for i in 1 2 3 4 5 6 7 8; do
    nanopolish eventalign --reads ./basecalled_fastq/sample$
{i}.fastq \
    --bam ./sample${i}_aligned_read_bam.fastq \
    --genome /path/to/reference-dna.fa \
    --print-read-names --scale-events --samples > sample${i}
_eventalign.tsv
done
```

```
# Collapse eventalign results using Nanocompore
for i in 1 2 3 4 5 6 7 8; do
    nanocompore eventalign_collapse --overwrite -t 6 \
    -i sample${i}_eventalign.tsv \
    -o sample${i}_eventalign_collapsed_reads.tsv &
done

# Perform differential analysis using Nanocompore
nanocompore sampcomp \
    --file_list1 sample1_eventalign_collapsed_reads.tsv/
out_eventalign_collapse.tsv, sample2_eventalign_collapsed_reads.tsv/
out_eventalign_collapse.tsv \
    --file_list2 sample3_eventalign_collapsed_reads.tsv/
out_eventalign_collapse.tsv, sample4_eventalign_collapsed_reads.tsv/
out_eventalign_collapse.tsv \
    --label1 noMTase \
    --label2 GII \
    --fasta /path/to/reference-dna.fa \
    --outpath results_GII

nanocompore sampcomp \
    --file_list1 sample1_eventalign_collapsed_reads.tsv/
out_eventalign_collapse.tsv, sample2_eventalign_collapsed_reads.tsv/
out_eventalign_collapse.tsv \
    --file_list2 sample5_eventalign_collapsed_reads.tsv/
out_eventalign_collapse.tsv, sample6_eventalign_collapsed_reads.tsv/
out_eventalign_collapse.tsv \
    --label1 noMTase \
    --label2 anc291 \
    --fasta /path/to/reference-dna.fa \
    --outpath results_anc291

nanocompore sampcomp \
    --file_list1 sample1_eventalign_collapsed_reads.tsv/
out_eventalign_collapse.tsv, sample2_eventalign_collapsed_reads.tsv/
out_eventalign_collapse.tsv \
    --file_list2 sample7_eventalign_collapsed_reads.tsv/
out_eventalign_collapse.tsv, sample8_eventalign_collapsed_reads.tsv/
out_eventalign_collapse.tsv \
    --label1 noMTase \
    --label2 SUPREM \
    --fasta /path/to/reference-dna.fa \
    --outpath results_SUPREM
```
