# Supplementary material for: SUPREM: an engineered non-site-specific m6A RNA methyltransferase with highly improved efficiency
Source: Nucleic Acids Res. 2024 Oct 17;52(20):12158–72. doi: 10.1093/nar/gkae887 (PMC11551740; doi:10.1093/nar/gkae887)
Supplement: gkae887_Supplemental_Files [file gkae887_supplemental_files.zip › SupplementaryTableS1.pdf]

Supplementary Table S1. Protein sequences of M.EcoGII and ancestral proteins

Name	Sequence
M.EcoGII	MLNTVKISSCELINADCLEFIRSLPENSVDLIVTDPPYFKVKPEGWDNQWKGDDDYLKWLDQCLAQFWRVLKPAGSLYLFCGHRLASDIEIMMRERFSVLNHIIWAKPSGRWNGCNKES<br>LRAYFPATERILFAEHYQGPYRPKDAGYEAKGRALKQHVMAPLIAYFRDARAALGITAKQIADATGKKNMVPHWFSASQWQLPNESDYLKLQSLFARVAEEKHQRGELEKPHHQLVST<br>YSELNRKYMELLSEYKNLRRYFGVTVQVPYTDVWTYKPVQYYPGKHPCEKPAEMLQQIISASSRPGDLVADFFMGSGSTVKAAMALGRRAIGVELETGRFEQTVREVQDLIV
Anc291	MKNTVKISSVELINADCLHYIQTLPDNSIDLIVTDPPYFKVKPNGWDNQWKGDEDYLKWLDQCLAQFWRVLKPAGSLYLFCGHRLASDIEIMMRERFNVLNHIIWAKPSGRWNGCNKES<br>LRAYFPATERILFAEHYQGPYRPKSDGYAAKGTELKQHVMAPLISYFRDARESLGVTSKQIAEATGKKNMVSHWFGASQWQLPNEADYQKLQALFTRIAAEKHQRNELEQPHHQLVAT<br>YHSLNRKYSELLEEYKSLRRYFSVSAAVPYTDVWTHKPVQFYPGKHPCEKPADMLRQIINASSRPGDLVADFFMGSGSTIKAAMALGRRAIGVELETERFNQTVSEIREL
Anc289	MKNTVKINSVELINADCLHYIATLPDNSIDLIVTDPPYFKVKPNGWDNQWKGDEDYLRWLDQCLAEFWRVLKPAGSLYLFCGHRLASDIEIMMRERFNVLNHIIWAKPSGRWNGCNKES<br>LRAYFPATERILFAEHYQGPYKPKSDGYAAKSTELKQHVMTPLISYFRDARESLGVTSKQIAEATGKKNMVSHWFGASQWQLPNEADYQKLQALFTRIAIEKHQRNELEQPHHQLVATY<br>QSLNRKYYELLEEYKSLRRYFSVSAAVPYTDVWTHKPVQFYPGKHPCEKPADMLRQIISASSRPGDVVADFFMGSGSTIKAAMELGRRAIGVELETERFNQTVSEIREL
Anc284 (SUPREM)	MKNTVNLNSINLVNADSLQYIKTLPDNCIDLIATDPPYFRVKSCAWDNQWENESAYLAWLDEVLAEFWRVLKPSGSLYMFCGSRLAADTELLMRERFNVLNHIIWAKPSGPWNRQNKES<br>LRAYFPATERILFAEHYQGPYKPKSSGYAVKCQELKQNVLKPLIDYFRNARQALGVSAKEIHAATGKKQMASHWFSESQWQLPNEEDYQKLQALFERIAAEKHQRNELSKPHHQLVKE<br>YQTLSRQYYELSQEYKSLRRPFSVTALVPYTDVWTYPPVQYYPGKHPCEKPAEMMRDIISASSRPGDVVADFFMGSGSTIKEAIKLGRRAIGVELEEERFNQTVSEIRAL
Anc250	MKNTVHLNSVQLVNADSLQYIKTLPDNSVDLIATDPPYFRVKSNAWDNQWESETAFLAWLDEFLAEFWRVLKPSGSLYMFCGSRLAADTELLVRQRFNVLNHIVWAKPSGPWRRCRK<br>ESLRSFFPATERIIFAEHYGAEGYAKGQSGYAAKCQELRQQVFKPLIDYFRNARQALGVSAKEINQATGKTQMCSHWFSESQWQLPSEEQYQKLQALFARKAAELGQAGALSKSHNE<br>LTKEYQALSRQYSGLVAQYDDLKAQYENLRRPFSVTKDVPYTDVWTFPPVQYYPGKHPCEKPAEMMEHIISASSRPGDVVADFFMGSGSTIKAALKLGRRAIGVELEEETFQQTVSEIE<br>DL
Anc242	MKNTLHNNSVTLVNADCLQYLKTLPDNSVDLILTDPPYFRVKSNAWDNQWESEESFLAWLDEVLVEFWRVLKPSGSLYLFCGSRLAADTELLIRQRFNVLNHIVWAKPSGPWRRCRKE<br>DLRSFFPSTERIIFAEHYGAEGFAKGSSGYATKCQELKQQVFKPLIDYFKNARESLGVSAKEINQATGTTQMCSHWFSYSQWQLPSEEQYQKLQALFAKKAAELGQAGELSKSHNELT<br>KEYQALHRQYQTLVRQYDDLKAQYENLRRPFSVTKDVPYTDVWTFPPVQYYPGKHPCEKPADMMEHIISASSRPGDVVLDAFMGSGSTGKACLKLGRRFIGIEMEEETFNQTVSSLE<br>DL
