# Supplementary material for: SUPREM: an engineered non-site-specific m6A RNA methyltransferase with highly improved efficiency
Source: Nucleic Acids Res. 2024 Oct 17;52(20):12158–72. doi: 10.1093/nar/gkae887 (PMC11551740; doi:10.1093/nar/gkae887)
Supplement: gkae887_Supplemental_Files [file gkae887_supplemental_files.zip › SupplementaryTableS3.pdf]

Supplementary Table S3. The initial velocity of DNA or RNA methylation reaction

	V_0 for DNA [x10^{-3} µM SAH/ µM adenosine/ min]	V_0 for RNA [x10^{-3} µM SAH/ µM adenosine/ min]
M.EcoGII	11.12 ± 1.45	0.12 ± 0.02
Anc291	27.94 ± 4.10	1.17 ± 0.04
SUPREM	9.72 ± 0.85	3.65 ± 0.63

The initial velocity was calculated based on Figure 1D and Supplementary Figure S4, normalized by the SAH calibration curve and concentration of adenosines in DNA or RNA. Each value represents the mean ± SEM of the three independent experiments.
