# Supplementary material for: SUPREM: an engineered non-site-specific m6A RNA methyltransferase with highly improved efficiency
Source: Nucleic Acids Res. 2024 Oct 17;52(20):12158–72. doi: 10.1093/nar/gkae887 (PMC11551740; doi:10.1093/nar/gkae887)
Supplement: gkae887_Supplemental_Files [file gkae887_supplemental_files.zip › SupplementaryTableS4.pdf]

Supplementary Table S4. Protein sequence of SUPREM variants

Name	Sequence
Anc284 (SUPREM)	MKNTVNLNSINLVNADSLQYIKTLPDNCIDLIATDPPYFRVKSCAWDNQWENESAYLAWLDEVLAEFWRVLKPSGSLYMFCGSRLAADTELLMRERFNVL<br>NHIIWAKPSGPWNRQNKESLRAYFPATERILFAEHYQGPYKPKSSGYAVKCQELKQNVLKPLIDYFRNARQALGVSAKEIHAATGKKQMASHWFSESQW<br>QLPNEEDYQKLQALFERIAAEKHQRNELSKPHHQLVKEYQTLSRQYYELSQEYKSLRRPFSVTALVPYTDVWTYPPVQYYPGKHPCEKPAEMMRDIISAS<br>SRPGDVVADFFMGSGSTIKEAIKLGRRAIGVELEEERFNQTVSEIRAL
SUPREM-S43P	MKNTVNLNSINLVNADSLQYIKTLPDNCIDLIATDPPYFRVK**P**CAWDNQWENESAYLAWLDEVLAEFWRVLKPSGSLYMFCGSRLAADTELLMRERFNVL<br>NHIIWAKPSGPWNRQNKESLRAYFPATERILFAEHYQGPYKPKSSGYAVKCQELKQNVLKPLIDYFRNARQALGVSAKEIHAATGKKQMASHWFSESQW<br>QLPNEEDYQKLQALFERIAAEKHQRNELSKPHHQLVKEYQTLSRQYYELSQEYKSLRRPFSVTALVPYTDVWTYPPVQYYPGKHPCEKPAEMMRDIISAS<br>SRPGDVVADFFMGSGSTIKEAIKLGRRAIGVELEEERFNQTVSEIRAL
SUPREM-C44N	MKNTVNLNSINLVNADSLQYIKTLPDNCIDLIATDPPYFRVKS**N**AWDNQWENESAYLAWLDEVLAEFWRVLKPSGSLYMFCGSRLAADTELLMRERFNVL<br>NHIIWAKPSGPWNRQNKESLRAYFPATERILFAEHYQGPYKPKSSGYAVKCQELKQNVLKPLIDYFRNARQALGVSAKEIHAATGKKQMASHWFSESQW<br>QLPNEEDYQKLQALFERIAAEKHQRNELSKPHHQLVKEYQTLSRQYYELSQEYKSLRRPFSVTALVPYTDVWTYPPVQYYPGKHPCEKPAEMMRDIISAS<br>SRPGDVVADFFMGSGSTIKEAIKLGRRAIGVELEEERFNQTVSEIRAL
SUPREM-A45G	MKNTVNLNSINLVNADSLQYIKTLPDNCIDLIATDPPYFRVKSN**G**WDNQWENESAYLAWLDEVLAEFWRVLKPSGSLYMFCGSRLAADTELLMRERFNVL<br>NHIIWAKPSGPWNRQNKESLRAYFPATERILFAEHYQGPYKPKSSGYAVKCQELKQNVLKPLIDYFRNARQALGVSAKEIHAATGKKQMASHWFSESQW<br>QLPNEEDYQKLQALFERIAAEKHQRNELSKPHHQLVKEYQTLSRQYYELSQEYKSLRRPFSVTALVPYTDVWTYPPVQYYPGKHPCEKPAEMMRDIISAS<br>SRPGDVVADFFMGSGSTIKEAIKLGRRAIGVELEEERFNQTVSEIRAL
SUPREM-A55D	MKNTVNLNSINLVNADSLQYIKTLPDNCIDLIATDPPYFRVKSCAWDNQWENES**D**YLAWLDEVLAEFWRVLKPSGSLYMFCGSRLAADTELLMRERFNVL<br>NHIIWAKPSGPWNRQNKESLRAYFPATERILFAEHYQGPYKPKSSGYAVKCQELKQNVLKPLIDYFRNARQALGVSAKEIHAATGKKQMASHWFSESQW<br>QLPNEEDYQKLQALFERIAAEKHQRNELSKPHHQLVKEYQTLSRQYYELSQEYKSLRRPFSVTALVPYTDVWTYPPVQYYPGKHPCEKPAEMMRDIISAS<br>SRPGDVVADFFMGSGSTIKEAIKLGRRAIGVELEEERFNQTVSEIRAL
SUPREM-E62Q	MKNTVNLNSINLVNADSLQYIKTLPDNCIDLIATDPPYFRVKSCAWDNQWENESAYLAWLD**Q**VLAEFWRVLKPSGSLYMFCGSRLAADTELLMRERFNVL<br>NHIIWAKPSGPWNRQNKESLRAYFPATERILFAEHYQGPYKPKSSGYAVKCQELKQNVLKPLIDYFRNARQALGVSAKEIHAATGKKQMASHWFSESQW<br>QLPNEEDYQKLQALFERIAAEKHQRNELSKPHHQLVKEYQTLSRQYYELSQEYKSLRRPFSVTALVPYTDVWTYPPVQYYPGKHPCEKPAEMMRDIISAS<br>SRPGDVVADFFMGSGSTIKEAIKLGRRAIGVELEEERFNQTVSEIRAL
SUPREM-S74A	MKNTVNLNSINLVNADSLQYIKTLPDNCIDLIATDPPYFRVKSCAWDNQWENESAYLAWLDEVLAEFWRVLKP**A**GSLYMFCGSRLAADTELLMRERFNVL<br>NHIIWAKPSGPWNRQNKESLRAYFPATERILFAEHYQGPYKPKSSGYAVKCQELKQNVLKPLIDYFRNARQALGVSAKEIHAATGKKQMASHWFSESQW<br>QLPNEEDYQKLQALFERIAAEKHQRNELSKPHHQLVKEYQTLSRQYYELSQEYKSLRRPFSVTALVPYTDVWTYPPVQYYPGKHPCEKPAEMMRDIISAS<br>SRPGDVVADFFMGSGSTIKEAIKLGRRAIGVELEEERFNQTVSEIRAL
SUPREM-S83H	MKNTVNLNSINLVNADSLQYIKTLPDNCIDLIATDPPYFRVKSCAWDNQWENESAYLAWLDEVLAEFWRVLKPSGSLYMFCG**H**RLAADTELLMRERFNVL<br>NHIIWAKPSGPWNRQNKESLRAYFPATERILFAEHYQGPYKPKSSGYAVKCQELKQNVLKPLIDYFRNARQALGVSAKEIHAATGKKQMASHWFSESQW<br>QLPNEEDYQKLQALFERIAAEKHQRNELSKPHHQLVKEYQTLSRQYYELSQEYKSLRRPFSVTALVPYTDVWTYPPVQYYPGKHPCEKPAEMMRDIISAS<br>SRPGDVVADFFMGSGSTIKEAIKLGRRAIGVELEEERFNQTVSEIRAL
SUPREM-T89I	MKNTVNLNSINLVNADSLQYIKTLPDNCIDLIATDPPYFRVKSCAWDNQWENESAYLAWLDEVLAEFWRVLKPSGSLYMFCGSRLAAD**I**ELLMRERFNVL<br>NHIIWAKPSGPWNRQNKESLRAYFPATERILFAEHYQGPYKPKSSGYAVKCQELKQNVLKPLIDYFRNARQALGVSAKEIHAATGKKQMASHWFSESQW<br>QLPNEEDYQKLQALFERIAAEKHQRNELSKPHHQLVKEYQTLSRQYYELSQEYKSLRRPFSVTALVPYTDVWTYPPVQYYPGKHPCEKPAEMMRDIISAS<br>SRPGDVVADFFMGSGSTIKEAIKLGRRAIGVELEEERFNQTVSEIRAL
SUPREM-P111R	MKNTVNLNSINLVNADSLQYIKTLPDNCIDLIATDPPYFRVKSCAWDNQWENESAYLAWLDEVLAEFWRVLKPSGSLYMFCGSRLAADTELLMRERFNVL<br>NHIIWAKPSG**R**WNRQNKESLRAYFPATERILFAEHYQGPYKPKSSGYAVKCQELKQNVLKPLIDYFRNARQALGVSAKEIHAATGKKQMASHWFSESQW<br>QLPNEEDYQKLQALFERIAAEKHQRNELSKPHHQLVKEYQTLSRQYYELSQEYKSLRRPFSVTALVPYTDVWTYPPVQYYPGKHPCEKPAEMMRDIISAS<br>SRPGDVVADFFMGSGSTIKEAIKLGRRAIGVELEEERFNQTVSEIRAL
SUPREM-R114G	MKNTVNLNSINLVNADSLQYIKTLPDNCIDLIATDPPYFRVKSCAWDNQWENESAYLAWLDEVLAEFWRVLKPSGSLYMFCGSRLAADTELLMRERFNVL<br>NHIIWAKPSGPWN**G**QNKESLRAYFPATERILFAEHYQGPYKPKSSGYAVKCQELKQNVLKPLIDYFRNARQALGVSAKEIHAATGKKQMASHWFSESQW<br>QLPNEEDYQKLQALFERIAAEKHQRNELSKPHHQLVKEYQTLSRQYYELSQEYKSLRRPFSVTALVPYTDVWTYPPVQYYPGKHPCEKPAEMMRDIISAS<br>SRPGDVVADFFMGSGSTIKEAIKLGRRAIGVELEEERFNQTVSEIRAL
SUPREM-Q115C	MKNTVNLNSINLVNADSLQYIKTLPDNCIDLIATDPPYFRVKSCAWDNQWENESAYLAWLDEVLAEFWRVLKPSGSLYMFCGSRLAADTELLMRERFNVL<br>NHIIWAKPSGPWNR**C**NKESLRAYFPATERILFAEHYQGPYKPKSSGYAVKCQELKQNVLKPLIDYFRNARQALGVSAKEIHAATGKKQMASHWFSESQW<br>QLPNEEDYQKLQALFERIAAEKHQRNELSKPHHQLVKEYQTLSRQYYELSQEYKSLRRPFSVTALVPYTDVWTYPPVQYYPGKHPCEKPAEMMRDIISAS<br>SRPGDVVADFFMGSGSTIKEAIKLGRRAIGVELEEERFNQTVSEIRAL
SUPREM-E179Q	MKNTVNLNSINLVNADSLQYIKTLPDNCIDLIATDPPYFRVKSCAWDNQWENESAYLAWLDEVLAEFWRVLKPSGSLYMFCGSRLAADTELLMRERFNVL<br>NHIIWAKPSGPWNRQNKESLRAYFPATERILFAEHYQGPYKPKSSGYAVKCQELKQNVLKPLIDYFRNARQALGVSAK**Q**IHAATGKKQMASHWFSESQW<br>QLPNEEDYQKLQALFERIAAEKHQRNELSKPHHQLVKEYQTLSRQYYELSQEYKSLRRPFSVTALVPYTDVWTYPPVQYYPGKHPCEKPAEMMRDIISAS<br>SRPGDVVADFFMGSGSTIKEAIKLGRRAIGVELEEERFNQTVSEIRAL
SUPREM-A182E	MKNTVNLNSINLVNADSLQYIKTLPDNCIDLIATDPPYFRVKSCAWDNQWENESAYLAWLDEVLAEFWRVLKPSGSLYMFCGSRLAADTELLMRERFNVL<br>NHIIWAKPSGPWNRQNKESLRAYFPATERILFAEHYQGPYKPKSSGYAVKCQELKQNVLKPLIDYFRNARQALGVSAKEIH**E**ATGKKQMASHWFSESQW<br>QLPNEEDYQKLQALFERIAAEKHQRNELSKPHHQLVKEYQTLSRQYYELSQEYKSLRRPFSVTALVPYTDVWTYPPVQYYPGKHPCEKPAEMMRDIISAS<br>SRPGDVVADFFMGSGSTIKEAIKLGRRAIGVELEEERFNQTVSEIRAL
SUPREM-K332A	MKNTVNLNSINLVNADSLQYIKTLPDNCIDLIATDPPYFRVKSCAWDNQWENESAYLAWLDEVLAEFWRVLKPSGSLYMFCGSRLAADTELLMRERFNVL<br>NHIIWAKPSGPWNRQNKESLRAYFPATERILFAEHYQGPYKPKSSGYAVKCQELKQNVLKPLIDYFRNARQALGVSAKEIHAATGKKQMASHWFSESQW<br>QLPNEEDYQKLQALFERIAAEKHQRNELSKPHHQLVKEYQTLSRQYYELSQEYKSLRRPFSVTALVPYTDVWTYPPVQYYPGKHPCEKPAEMMRDIISAS<br>SRPGDVVADFFMGSGSTIKEAI**A**LGRRAIGVELEEERFNQTVSEIRAL

A red and bold letter represented a mutation site.
