# Supplementary material for: SUPREM: an engineered non-site-specific m6A RNA methyltransferase with highly improved efficiency
Source: Nucleic Acids Res. 2024 Oct 17;52(20):12158–72. doi: 10.1093/nar/gkae887 (PMC11551740; doi:10.1093/nar/gkae887)
Supplement: gkae887_Supplemental_Files [file gkae887_supplemental_files.zip › SupplementaryTableS5.pdf]

Supplementary Table S5. DALI result for M.EcoGII target recognition domains

PDB-ID	Z-score	RMSD	Name	Function
7p4a	6.5	3.2	NON-CANONICAL STAPHYLOCOCCUS AUREUS PATHOGENICITY ISLAND REPRESSION.	DNA BINDING PROTEIN
2h8r	5.9	2.2	HEPATOCYTE NUCLEAR FACTOR 1-BETA	TRANSCRIPTION ACTIVATOR/DNA
7t8i	5.8	2.1	THE IMMR TRANSCRIPTIONAL REGULATOR DNA-BINDING 2 DOMAIN OF BACILLUS SUBTILIS	DNA BINDING PROTEIN
3ivp	5.8	2	A POSSIBLE TRANSPOSON-RELATED DNA-BINDING PROTEIN 2 FROM CLOSTRIDIUM DIFFICILE 630	DNA BINDING PROTEIN
3omt	5.8	2.2	UTATIVE ANTITOXIN COMPONENT	UNKNOWN FUNCTION
7t5u	5.8	2.2	E. COLI MS115-1 CAPH N-TERMINAL DOMAIN	DNA BINDING PROTEIN
6jq1	5.8	2.2	DDRO FROM DEINOCOCCUS GEOTHERMALIS	DNA BINDING PROTEIN
1utx	5.7	2.4	REGULATION OF CYTOLYSIN EXPRESSION BY ENTEROCOCCUS FAECALIS	DNA BINDING PROTEIN
4yba	5.7	2.2	THE C.KPN2I CONTROLLER PROTEIN	GENE REGULATION
3f51	5.6	2.4	THE CLP GENE REGULATOR CLGR FROM CORYNEBACTERIU	TRANSCRIPTION ACTIVAT
